# Targeted sequencing identifies the mutational signature of double primary and metastatic malignancies: a case report

**DOI:** 10.1186/s13000-019-0874-5

**Published:** 2019-09-04

**Authors:** Chuangzhou Rao, Liangqin Nie, Xiaobo Miao, Analyn Lizaso, Guofang Zhao

**Affiliations:** 1 Department of Radiotherapy and Chemotherapy, Hwamei Hospital, University of Chinese Academy Of Sciences, No.41 Northwest Street, Haishu District, Ningbo, 315010 Zhejiang China; 2 Burning Rock Biotech, Guangzhou, 510300 China; 3 Department of Cardiothoracic Surgery, Hwamei Hospital, University of Chinese Academy of Sciences, No. 41 Northwest Street, Haishu District, Ningbo, 315010 Zhejiang China

**Keywords:** Tissue of origin, Mutational profiling, Multiple primary tumors

## Abstract

**Background:**

The accurate identification of the tissue of origin is critical for optimal management of cancer patients particularly those who develop multiple malignancies; however, conventional diagnostic methods at times may fail to provide conclusive diagnosis of the origin of the malignancy. Herein, we describe the use of targeted sequencing in distinguishing the primary and metastatic tumors in a patient with metachronous malignancies in the lung, colon and kidney.

**Case presentation:**

In December 2016, a 55-year-old Chinese male was diagnosed with stage IB lung adenosquamous carcinoma and treated with left lower lobectomy and 4 cycles of platinum-based chemotherapy. After being disease-free for 3.5 months, three colonic polyps were discovered and were diagnosed as invasive adenocarcinoma after polypectomy. Within 5.4 months from the polypectomy, squamous cell renal carcinoma was identified and was managed by radical nephrectomy. Immunohistochemistry results were inconclusive on the origin of the kidney tumor. Hence, the three archived surgical tissue samples were sequenced using a targeted panel with 520 cancer-related genes. Analysis revealed similar mutational signature between the lung and kidney tumors and a distinct mutational profile for the colon tumor, suggesting that the lung and colon malignancies were primary tumors, while the kidney tumor originated from the lung, revealing a diagnosis of metastatic double primary cancer – lung carcinoma with renal cell metastasis and second primary colon carcinoma.

**Conclusion:**

Mutational profiling using targeted sequencing is valuable not only for the detection of actionable mutations, but also in the identification of the origin of tumors. This diagnostic approach should be considered in similar scenarios.

**Electronic supplementary material:**

The online version of this article (10.1186/s13000-019-0874-5) contains supplementary material, which is available to authorized users.

## Background

The accurate diagnosis of the tissue of origin and cancer type is critical in guiding the optimal management and improving the treatment outcome of cancer patients with multiple malignancies. The standard method to determine the tissue of origin involves pathologic evaluation of tumor samples using histologic examination and selected panels of immunohistochemical (IHC) stains combined with clinical evaluation of the patient including history taking, physical examination, clinical chemistry and medical imaging. Despite the use of exhaustive antibody panels in IHC, histopathology-based diagnosis is only conclusive in 34 to 69% of the cases [1–7]. On the contrary, numerous studies have consistently demonstrated between 75 to 90% accuracy in identifying the tissue of origin by the analysis of gene expression, indicating the superiority of molecular profiling compared to IHC in the identification of the primary tumor [1–7]. Consistent with gene expression analysis, mutational profiling can also identify molecular signatures from tissues and distinguish between primary and metastatic tumors from various organs [8–12]. Although next-generation sequencing (NGS)-based mutational profiling has been integrated into the routine clinical practice for therapeutic guidance, it is still not widely used in the identification of the tissue of origin particularly in patients with suspected multiple primary cancers. Herein, we describe the application of capture-based targeted sequencing in distinguishing the origin of the metachronous malignancies in the lung, colon and kidney of our patient.

## Case presentation and management

**Fig. 1:**
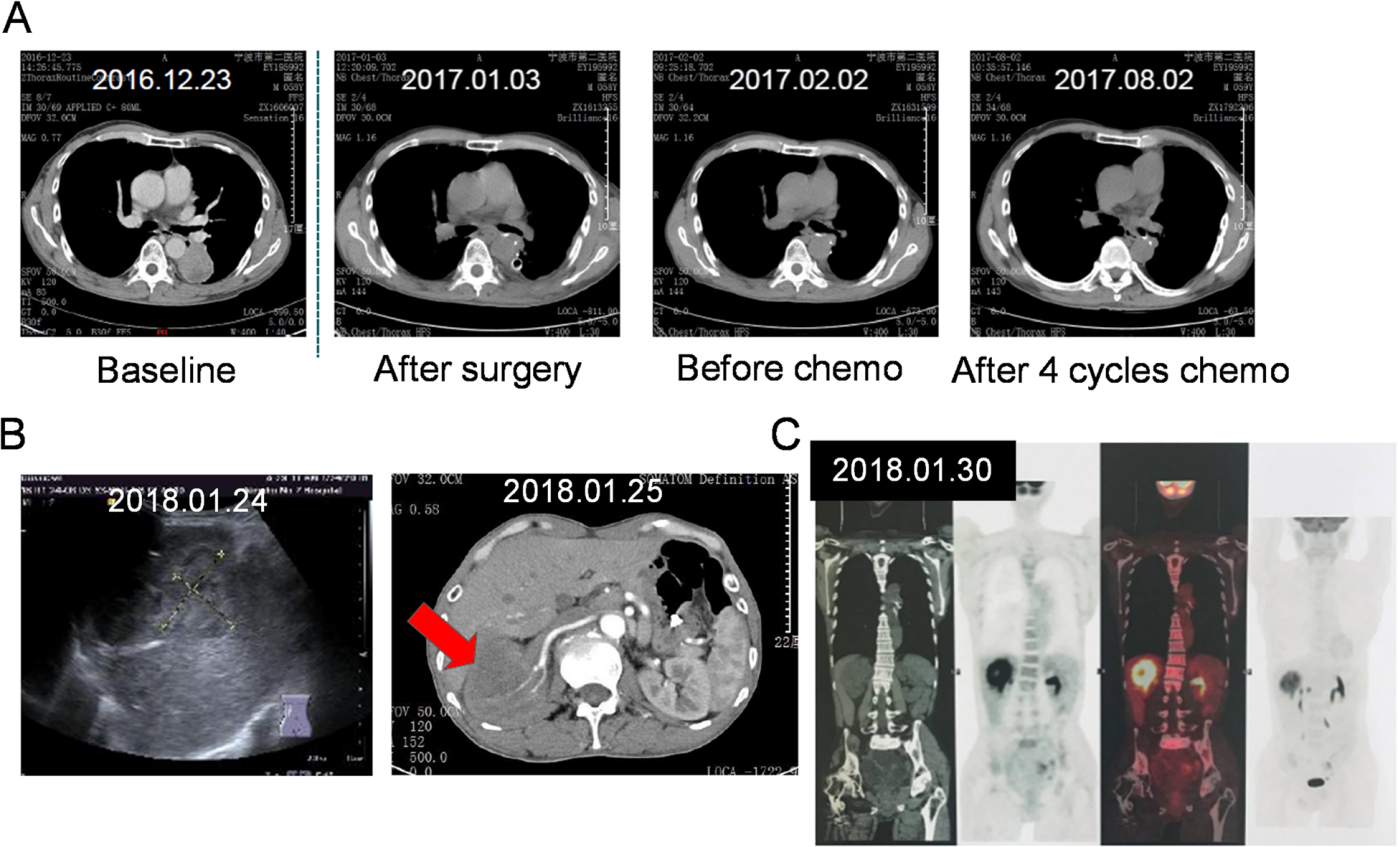
**a** Thoracic CT scan illustrating the tumor on the left lower lung and successful treatment with lobectomy and 4 cycles of platinum-based chemotherapy. **b** Ultrasonography and CT urography scans illustrating the detection of kidney tumor on January 2018. **c** PET/CT scans illustrating the increased FDG metabolism on the right kidney and colon, and the absence of disease recurrence or relapse in the lung

**Fig. 2:**
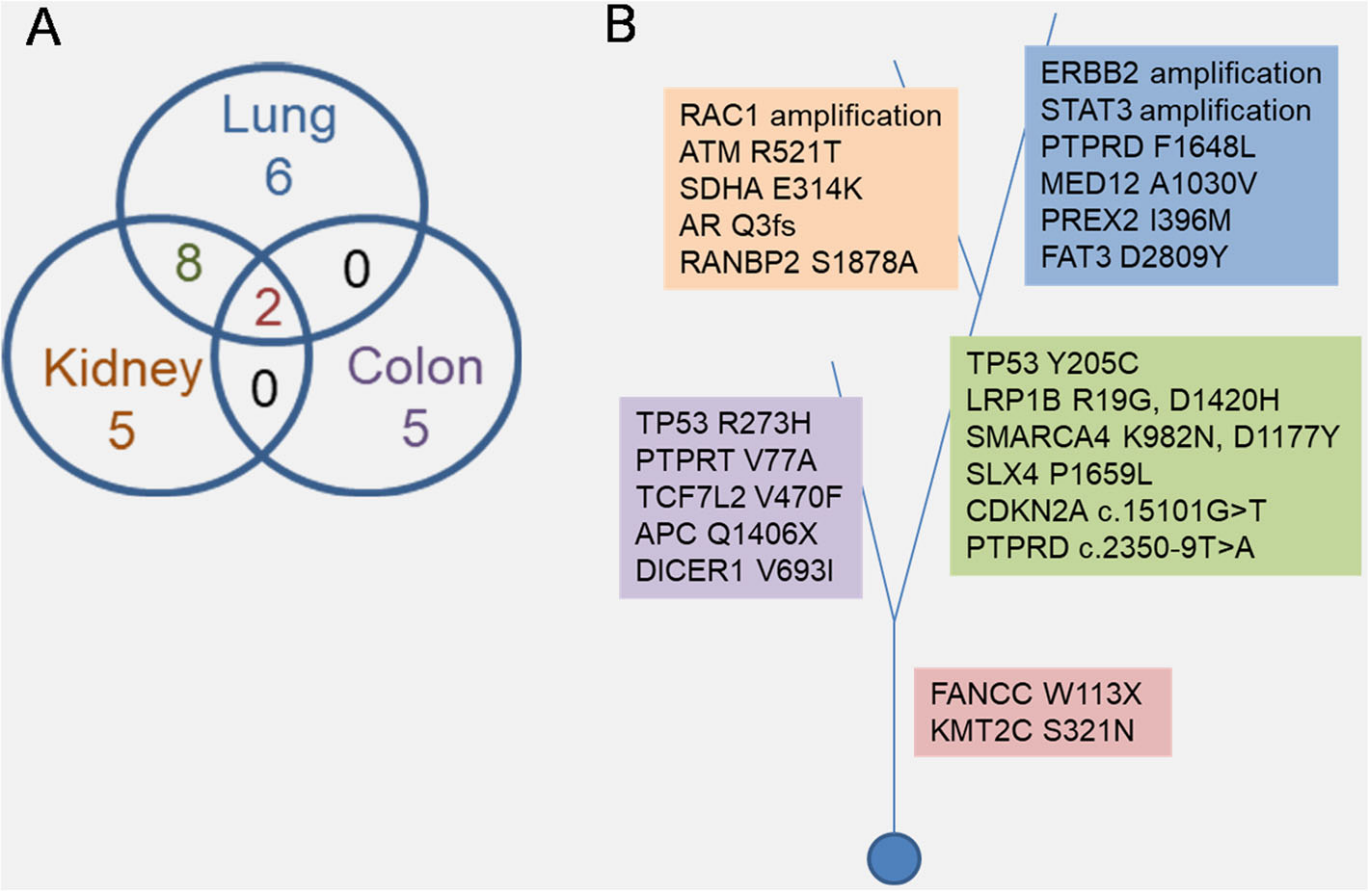
Mutations detected among the lung, kidney and colon tumors. **a** Venn diagram illustrating the number of shared and specific mutations among the tumors. **b** Cladogram illustrating the mutations shared and distinct to each of the tumors. Shading in pink denotes mutations shared among the three tumor samples; Green for mutations shared between the lung and kidney samples; Blue for lung-specific mutations; Orange for kidney-specific mutations and Purple denotes colon-specific mutations

**Fig. 3:**
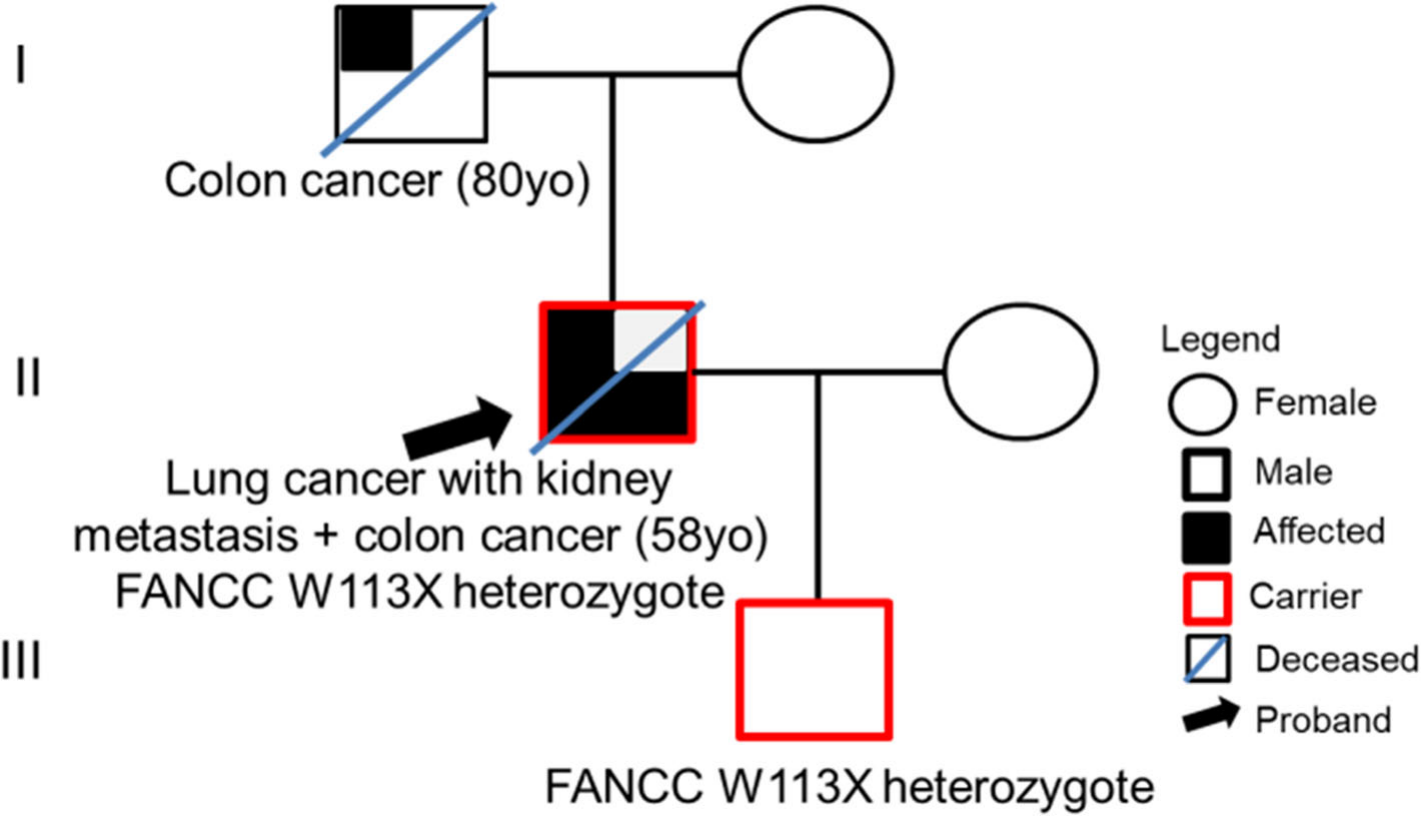
Pedigree analysis illustrating the family history of colon cancer and the detection of FANCC W113X in the patient (II) and his son (III)

On December 2016, a 55-year-old, non-smoker Chinese male with an ECOG score of 0 underwent chest X-ray due to fracture of the left clavicle. The chest X-ray incidentally revealed a space-occupying lesion in the left lower lobe of the lung. Except for persistent coughing for a month and mild hemoptysis, no other clinical symptoms were reported by the patient. Thoracic computed tomography (CT) scans confirmed the chest X-ray results showing a tumor volume of 41x44mm with a relatively clear margin. The disease was pathologically staged as T2aN0M0 (stage IB) after left lower lobectomy and mediastinoscopy. Further histologic examination of the surgical tissues revealed adenosquamous lung carcinoma with predominantly papillary adenocarcinoma pattern (Additional file 1: Figure S1). Adjuvant chemotherapy was also administered to the patient with a total of 4 cycles of chemotherapy regimen including 2 cycles of 40 mg vinorelbine plus 80 mg nedaplatin and 2 cycles of 100 mg docetaxel plus 80 mg nedaplatin administered every 3 weeks. On August 2017, chest CT revealed no disease recurrence in the lungs (Fig. 1a); however, colonic polyps were discovered during his physical examination. Initial biopsy revealed tubulovillous adenoma with low-grade intraepithelial neoplasia. Complete endoscopic resection was scheduled and removed three polyps in the transverse colon with sizes between 6 to 8 mm. Histopathologic analyses revealed moderately differentiated adenocarcinoma with submucosal invasion. No systemic therapy was administered to the patient. On December 2017, after being disease-free for about 5.4 months, the patient returned to the clinic due to pain and discomfort in the right waist. Ultrasonography and CT urography revealed moderately increased renal echogenicity and a 51x60x68mm obstruction in the right kidney, respectively, which are highly suggestive of renal carcinoma (Fig. 1b). In addition to revealing an elevated 2-[^18^F]-fluoro-2-deoxy-D-glucose (FDG) uptake of the right kidney that is indistinguishable from the liver, positron-emission tomography (PET)/CT scanning also revealed mediastinal lymphadenopathy and slightly elevated FDG uptake of the sigmoid colon, suggestive of malignancy and metastasis (Fig. 1c). No sign of disease recurrence or relapse was detected in the thoracic region (Fig. 1c). Right radical nephrectomy and inferior vena cava thrombectomy were performed to remove the 55x53x35mm tumor. Further examination revealed renal pelvis invasion with renal vein involvement and presence of a 13 mm nodule in the renal hilum. No growth was found in the renal capsule and ureteral muscle. Histopathology results revealed poorly differentiated tumor cells. The new malignancy was suspected to be squamous cell urothelial carcinoma, but with the possibility of kidney metastasis considering a history of lung cancer. However, the lack of immunoreactivity of the kidney tumor sample with thyroid transcription factor 1 (TTF-1) and cytokeratin 7 (CK-7) antibodies did not support pulmonary origin (Table 1, Additional file 1: Figure S2). To rule out the possibility of the tumor in the kidney being another primary site, we explored the use of capture-based targeted NGS to understand its biology compared to the two earlier malignancies. The three archived surgical tissues, as well as paired white blood cell samples, were sequenced using a targeted panel with 520 cancer-related genes, spanning 1.64 Mb of the human genome (OncoScreen Plus, Burning Rock Biotech). A total of 16, 15 and 7 mutations were detected in the lung, kidney and colon tumor samples, respectively (Table 2). Interestingly, comparative analysis revealed a similar mutational signature between the lung and kidney tumors, sharing 67% (10/15) of the mutations. Except for the FANCC W113X and KMT2C S321 N common to the three tumor samples, no other mutations were shared between the colon and either the lung or the kidney tumor samples (Fig. 2a). These sequencing results indicate that both the lung and colon tumors were primary tumors; while the kidney tumor originated from the lung (Fig. 2b). Consistent with the detection of FANCC W113X in all the three tumors, the pathogenic mutation was also detected in his germline as a heterozygous mutation (Table 2). In addition, FANCC W113X was also detected in the son of the patient (III, Fig. 3). Further investigation of the family history revealed that the father of the patient was previously treated for colon cancer with no recurrence reported (I, Fig. 3). After the nephrectomy, the patient did not receive further systemic treatment due to persistent fever and infection. The patient passed away on August 2018 due to severe infection, with an overall survival of 20.4 months. His son is still currently cancer-free.

**Table 1:** Immunohistochemistry results for the lung and kidney tumors

Antibodies used for IHC	Lung	Kidney
Cytokeratin 5/6 (CK-5/6)	+	+++
Cytokeratin 7 (CK-7)	++	–
Thyroid transcription factor − 1 (TTF-1)	+++	–
Cytokeratin 20 (CK-20)	–	–
CDX-2	NA	–
NapsinA	+++	–
Ki-67	20%	60%
P40	+	NA
P63	NA	+++
PAX-8	NA	–
Vimentin	–	NA
CD56	–	NA
S-100	NA	–

Note: +, positive immunoreactivity; −, negative result; NA, not applicable/tested

**Table 2:** Gene mutations detected in the lung, kidney and colon tumor samples

	Lung	Kidney	Colon	White blood cell
Tumor Mutation Burden (mutations/Mb)	15.1	14.3	7.9	–
Gene mutations detected	Allele frequency			
FANCC c.339G > A (p.W113X)	36.45%	39.67%	46.57%	detected
KMT2C c.962G > A (p.S321 N)	11.81%	14.62%	17.45%	–
TP53 c.614A > G (p.Y205C)	31.74%	24.91%	–	–
CDKN2A c.151-1G > T	24.40%	15.54%	–	–
LRP1B c.55A > G (p.R19G)	49.76%	46.88%	–	–
LRP1B c.4258G > C (p.D1420H)	17.64%	15.00%	–	–
SMARCA4 c.2946G > T (p.K982 N)	30.56%	26.12%	–	–
SMARCA4 c.3529G > T (p.D1177Y)	26.92%	27.18%	–	–
SLX4 c.4976C > T (p.P1659L)	22.82%	6.83%	–	–
PTPRD c.2350-9 T > A	22.21%	18.26%	–	–
PTPRD c.4944 T > A (p.F1648 L)	9.66%	–	–	–
MED12 c.3089C > T (p.A1030V)	13.64%	–	–	–
PREX2 c.1188C > G (p.I396M)	12.02%	–	–	–
FAT3 c.8425G > T (p.D2809Y)	7.25%	–	–	–
ERBB2 amplification	CN = 3.38	–	–	–
STAT3 amplification	CN = 3.66	–	–	–
RAC1 amplification	–	CN = 3.07	–	–
ATM c.1562G > C (p.R521T)	–	8.04%	–	–
SDHA c.940G > A (p.E314K)	–	13.71%	–	–
ATR c.7del (p.Q3fs)	–	8.88%	–	–
RANBP2 c.5632 T > G (p.S1878A)	–	4.47%	–	–
TP53 c.818G > A (p.R273H)	–	–	16.77%	–
PTPRT c.230 T > C (p.V77A)	–	–	29.21%	–
TCF7L2 c.1408G > T (p.V470F)	–	–	15.71%	–
APC c.4216C > T (p.Q1406X)	–	–	14.99%	–
DICER1 c.2077G > A (p.V693I)	–	–	14.86%	–

## Discussion

Multiple primary cancer (MPC) is defined as the simultaneous (synchronous) or successive (metachronous) development of independent primary malignant tumors in one or multiple organs in the same individual. The development of MPC can be due to common environmental risk factors or germline mutations, with an incidence between 2.4 to 17% [13, 14]. According to a retrospective study involving Chinese lung cancer patients, 3.4% of these patients develop MPC, with colorectal, esophageal and thyroid cancers as the most frequent second primary tumor [15]. Interestingly, patients who developed metachronous MPC had significantly better overall survival (OS) than those who developed synchronous MPC (median OS: 72.8 months vs. 12.9 months, *P* < 0.001) [15]. Although the patient we described in this report was fortunate with the repeated incidental discovery of his lung and colon tumors at their early stage, he only survived less than 2 years from the time of the diagnosis of the first cancer. We speculate that the development of multiple malignancies in our patient and his poor prognosis were due to the heterozygous germline FANCC mutation. The pathogenic FANCC mutation was consistently detected in all the somatic tissues tested and his germline and was also detected in his son. Despite the refusal of the father of the patient to undergo genetic testing, his history of colon cancer and the detection of the same FANCC mutation in the patient’s son strongly suggest a genetic component in the development of the malignancies in this patient. The particular FANCC mutation detected in the patient, c.339G > A (p.W113X), is recorded as pathogenic in ClinVar database (ID: 370175). Mutations in Fanconi complementation group C (FANCC) is associated with the development of Fanconi anemia (FA), an autosomal recessive disease characterized by developmental abnormalities, chromosomal instability and cancer susceptibility. While homozygous germline FA gene mutations increased the risk for inherited cancer predisposition of individuals affected with Fanconi anemia [16], heterozygous carriers of germline FANCC mutations, albeit having been documented in only a few patients, have also increased susceptibility to develop certain cancers including pancreatic, colorectal, breast and lung cancers [16–22].

As the treatment improves and becomes more type-specific for many cancers, the need for the identification of the tissue of origin of the tumor becomes more important than ever for the selection of optimal management. IHC, as a gold standard, is used in the routine assessment of tumor samples for diagnostic purpose. However, in some cases, as in our patient, IHC was only suggestive of the diagnosis and did not provide a conclusive origin of the malignancy. The kidney tumor of our patient, although the lack of immunoreactivity for CK-20 and CDX-2 ruled out the possibility of metastasis from intestinal origin, the negative results with TTF-1 and CK-7 was not supportive of pulmonary origin (Table 1, Additional file 1: Figure S2). CK-20 and CDX-2 are markers commonly associated with colonic adenocarcinoma, while TTF-1 and CK-7 are markers of pulmonary epithelial cells and pulmonary adenocarcinoma, respectively [23, 24]. The IHC results from the lung and kidney tumors (Additional file 1: Figure S1-S2) and the lack of disease recurrence in the lung raises the possibility that the tumor in the kidney could be another primary. To further explore the origin of the kidney tumor and shed light whether it is another primary or a metastatic tumor, we submitted the archived surgical tissues of the three malignancies for comprehensive mutational profiling using a panel with 520 cancer-related genes. Genomic methods including comparative genomic hybridization (CGH), PCR, Sanger sequencing and NGS has been used for the mutational profiling of various tumors to identify the primary and metastatic tumors [8–12]. Consistent with the findings from Becerra et al. [12], some mutations are clonal which are shared between the primary and metastatic tumors; however, other mutations are subclonal and can only be found in either primary or metastatic tumors. The clonal and subclonal relationship among the liver, kidney and colon malignancies has been illustrated in Fig. 2c and demonstrates that lung and colon are primary tumors, sharing only the two clonal mutations; while the kidney tumor originated from the lung, sharing a total of 10 mutations. Despite the lack of IHC results for the colon tumor due to limited sample amount, NGS was able to identify it as a primary tumor with its distinct mutational profile from the two other malignancies. The use of NGS-based mutational profiling, not only reveals actionable mutations, but also mutational signatures of primary and metastatic tumor samples allowing their distinction Although still limited by cost and the requirement for highly-trained personnel, this approach has the potential to improve diagnosis and reduce the risk of misdiagnosing the metastasis as a primary or vice versa. Another advantage of NGS is the requirement for smaller amount of samples, which is usually a limiting factor in IHC that requires multiple tissue sections to test antibody panels individually. It has to be noted that mutational profiling should be coupled with standard pathological tests.

## Conclusion

Our case demonstrates the efficient use of capture-based targeted sequencing to elucidate the mutational profile of the tumors and identify the origin of a metastatic malignancy which was otherwise diagnosed as another primary tumor by IHC. In certain scenarios where it is difficult to establish the origin of an additional malignancy, mutational profiling can provide guidance on the origin of the tumor. We therefore support the incorporation of mutational profiling as a diagnostic approach in identifying the tissue of origin of tumor samples to complement the standard pathology test.

## Additional files

Additional file 1: **Figure S1**. Histologic features of the lung tumor sample of the patient. A. Hematoxylin-eosin (HE) stain (magnification X200); B-O. Immunohistochemical staining of B. thyroid transcription factor 1 (TTF1); C. Napsin A; D. cytokeratin-7 (CK7); E. cytokeratin 5/6 (CK5/6); F.cytokeratin-20 (CK20); G. carcinoembyonic antigen (CEA); H. EGFR; I. Ki-67; J. GATA3; K. P40; L. Vimentin; M. estrogen receptor (ER); N. progesterone receptor (PR); O. synaptophysin (syn) (magnification X40). **Figure S2**. Histologic features of the kidney tumor sample of the patient. A. Hematoxylin-eosin (HE) stain (magnification X200); B-L. Immunohistochemical staining of B. thyroid transcription factor 1 (TTF1) (magnification X100); C. Napsin A; D. cytokeratin-7 (CK7); E. cytokeratin 5/6 (CK5/6); F.cytokeratin-20 (CK20);G. pan-cytokeratin (CKPan); H. P63; I. Ki67; J. PAX-8; K S-100; *L. Melan* A (magnification X40) (PDF 1018 kb)

## Data Availability

The dataset generated and analyzed within this report is available from the corresponding author upon reasonable request.

## Abbreviations

CT: Computed tomography
ECOG: Eastern Cooperative Oncology Group;
FDG: 2-[^18^F]-fluoro-2-deoxy-D-glucose
IHC: Immunohistochemistry
MPC: Multiple primary cancers
PET: Positron emission tomography
TNM: Tumor node metastasis
