# Supplementary material for: Targeted sequencing identifies the mutational signature of double primary and metastatic malignancies: a case report
Source: Diagn Pathol. 2019 Sep 4;14:101. doi: 10.1186/s13000-019-0874-5 (PMC6727526; doi:10.1186/s13000-019-0874-5)
Supplement: Supplementary file 1 — Figure S1. Histologic features of the lung tumor sample of the patient. A. Hematoxylin-eosin (HE) stain (magnification X200); B-O. Immunohistochemical staining of B. thyroid transcription factor 1 (TTF1); C. Napsin A; D. cytokeratin-7 (CK7); E. cytokeratin 5/6 (CK5/6); F.cytokeratin-20 (CK20); G. carcinoembyonic antigen (CEA); H. EGFR; I. Ki-67; J. GATA3; K. P40; L. Vimentin; M. estrogen receptor (ER); N. progesterone receptor (PR); O. synaptophysin (syn) (magnification X40). Figure S2. Histologic features of the kidney tumor sample of the patient. A. Hematoxylin-eosin (HE) stain (magnification X200); B-L. Immunohistochemical staining of B. thyroid transcription factor 1 (TTF1) (magnification X100); C. Napsin A; D. cytokeratin-7 (CK7); E. cytokeratin 5/6 (CK5/6); F.cytokeratin-20 (CK20);G. pan-cytokeratin (CKPan); H. P63; I. Ki67; J. PAX-8; K S-100; L. Melan A (magnification X40) (PDF 1018 kb) [file 13000_2019_874_MOESM1_ESM.pdf]

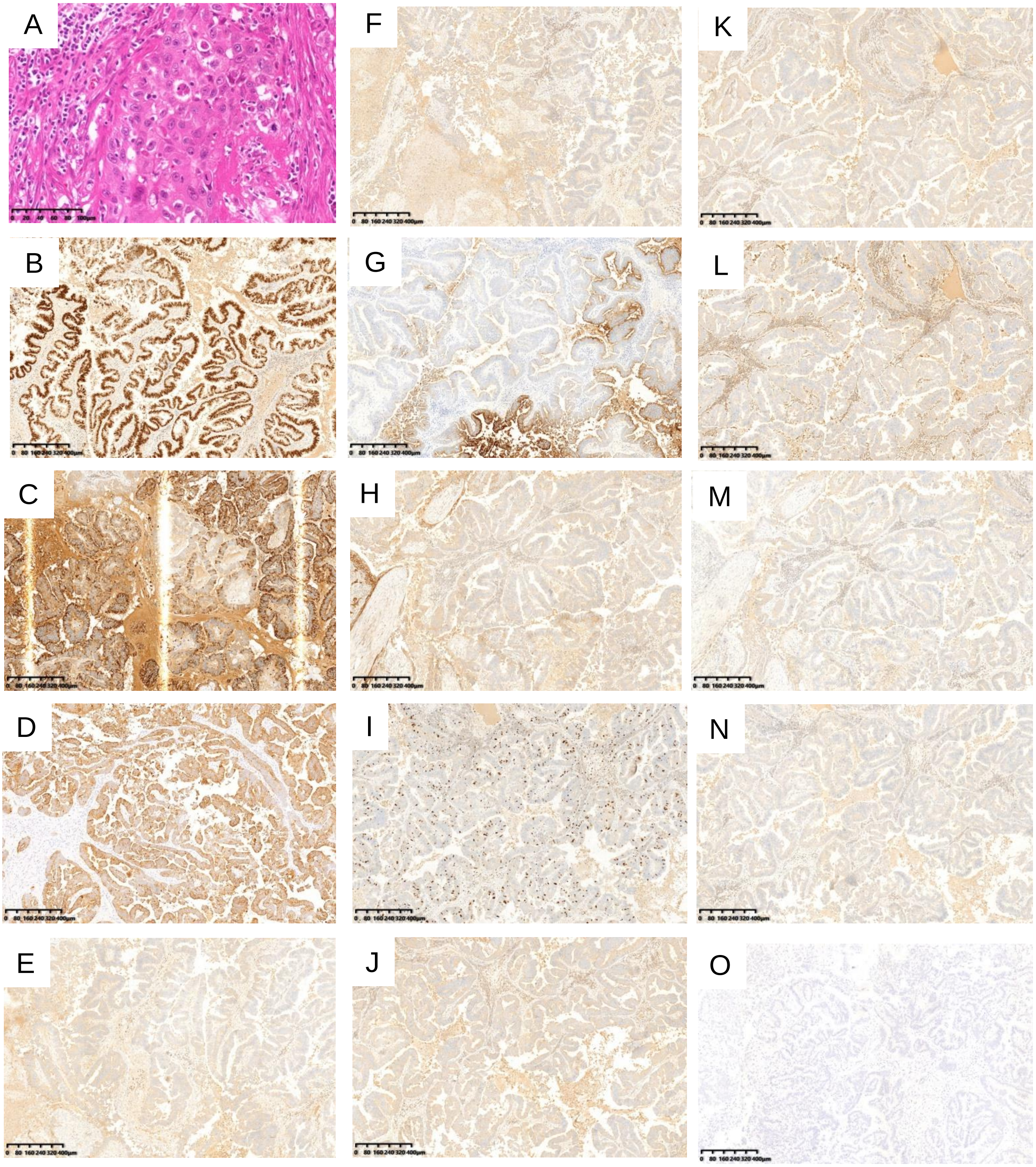


Figure S1. Histologic features of the lung tumor sample of the patient. A. Hematoxylin-eosin (HE) stain (magnification X200); B-O. Immunohistochemical staining of B. thyroid transcription factor 1 (TTF1); C. Napsin A; D. cytokeratin-7 (CK7); E. cytokeratin 5/6 (CK5/6); F.cytokeratin-20 (CK20); G. carcinoembyonic antigen (CEA); H. EGFR; I. Ki-67; J. GATA3; K. P40; L. Vimentin; M. estrogen receptor (ER); N. progesterone receptor (PR); O. synaptophysin (syn) (magnification X40)

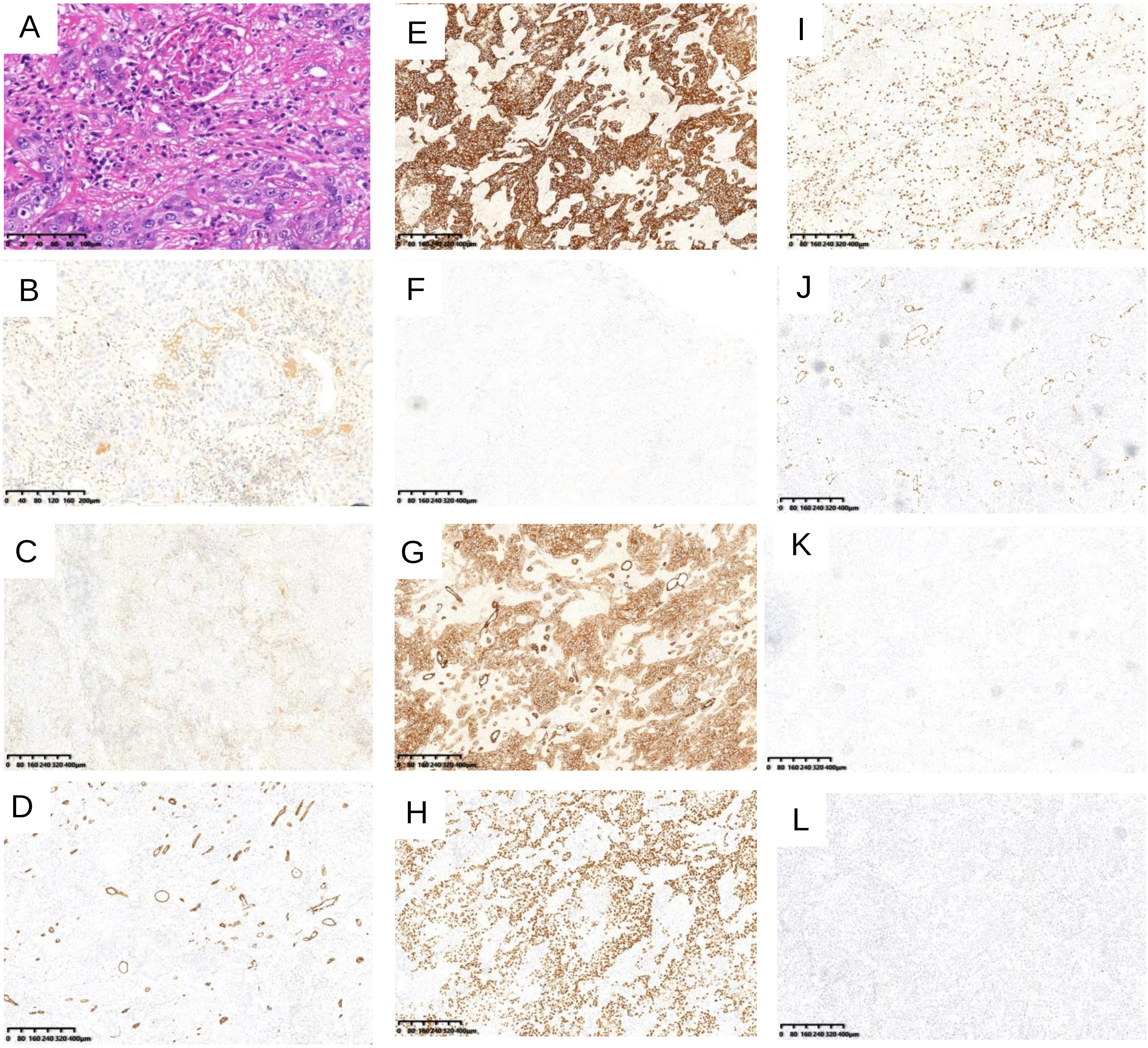


Figure S2. Histologic features of the kidney tumor sample of the patient. A. Hematoxylin-eosin (HE) stain (magnification X200); B-L. Immunohistochemical staining of B. thyroid transcription factor 1 (TTF1) (magnification X100); C. Napsin A; D. cytokeratin-7 (CK7); E. cytokeratin 5/6 (CK5/6); F.cytokeratin-20 (CK20);G. pan-cytokeratin (CKPan); H. P63; I. Ki67; J. PAX-8; K S-100; L. Melan A (magnification X40)
